# Characterization of putative drug resistant biomarkers in *Plasmodium falciparum* isolated from Ghanaian blood donors

**DOI:** 10.1186/s12879-020-05266-2

**Published:** 2020-07-22

**Authors:** Enoch Aninagyei, Kwabena Obeng Duedu, Tanko Rufai, Comfort Dede Tetteh, Margaretta Gloria Chandi, Paulina Ampomah, Desmond Omane Acheampong

**Affiliations:** 1 Department of Biomedical Sciences, School of Basic and Biomedical Sciences, University of Health and Allied Sciences, Ho, Volta Region Ghana; 2 Ghana Health Service, Accra, Ghana; 3 New Juabeng Municipal Health Directorate, Koforidua, Ghana; 4 Ghana Health Service, Municipal Health Directorate, Ga West Municipal, Amasaman Ghana; 5 Ga North Municipal Health Directorate, Ofankor-Accra, Greater Accra Region, Ghana; 6 School of Allied Health Sciences, Department of Biomedical Sciences, School of Allied Health Sciences, College of Health and Allied Science, University of Cape Coast, Cape Coast, Ghana; 7 Malaria Genome Laboratory, Wellcome Sanger Institute, Hinxton, Cambridgeshire CB10 1SA UK

**Keywords:** *Plasmodium falciparum*, Blood donors, Putative drug resistant biomarkers, Mutant haplotypes, Ghana

## Abstract

**Background:**

*Plasmodium falciparum* parasites, which could harbour anti-malaria drug resistance genes, are commonly detected in blood donors in malaria-endemic areas. Notwithstanding, anti-malaria drug resistant biomarkers have not been characterized in blood donors with asymptomatic *P. falciparum* infection.

**Methods:**

A total of 771 blood donors were selected from five districts in the Greater Accra Region, Ghana. Each donor sample was screened with malaria rapid diagnostic test (RDT) kit and parasitaemia quantified microscopically. Dried blood spots from malaria positive samples were genotyped for *P. falciparum* chloroquine resistance transporter (*Pfcrt*), *P. falciparum* multi-drug resistance (*Pfmdr1*), *P. falciparum* dihydropteroate-synthetase (*Pfdhps*), *P. falciparum* dihydrofolate-reductase (*Pfdhfr*) and *Kelch 13* propeller domain on chromosome 13 (*Kelch 13*) genes.

**Results:**

Of the 771 blood donors, 91 (11.8%) were positive by RDT. Analysis of sequence reads indicated successful genotyping of *Pfcrt*, *Pfmdr1*, *Pfdhfr*, *Pfdhps* and *Kelch 13* genes in 84.6, 81.3, 86.8, 86.9 and 92.3% of the isolates respectively. Overall, 21 different mutant haplotypes were identified in 69 isolates (75.8%). In *Pfcrt*, CVIET haplotype was observed in 11.6% samples while in *Pfmdr1,* triple mutation (resulting in YFN haplotype) was detected in 8.1% of isolates. In *Pfdhfr* gene, triple mutation resulting in IRNI haplotype and in *Pfdhps* gene, quintuple mutation resulting in AGESS haplotype was identified in 17.7% parasite isolates. Finally, five non-synonymous *Kelch 13* alleles were detected; C580Y (3.6%), P615L (4.8%), A578S (4.8%), I543V (2.4%) and A676S (1.2%) were detected.

**Conclusion:**

Results obtained in this study indicated various frequencies of mutant alleles in *Pfcrt*, *Pfmdr1*, *Pfdhfr*, *Pfdhps* and *Kelch 13* genes from *P. falciparum* infected blood donors. These alleles could reduce the efficacy of standard malaria treatment in transfusion-transmitted malaria cases. Incorporating malaria screening into donor screening protocol to defer infected donors is therefore recommended.

## Background

Lack of sensitive screening tools for detecting asymptomatic malaria in blood donors is believed to have contributed significantly to the underestimation of the global burden of transfusion-transmitted malaria (TTM) [1]. In Ghana, the prevalence of malaria among blood donors have been reported to be between 8 and 13.7% by rapid diagnostic testing (RDT) and 3–4.7% by microscopy [2–4]. In 2016, cases of TTM were reported from different provinces in Iran [5] and in Ghana [2]. In blood recipients with no or reduced immunity to malaria, TTM can be fatal, if infections are not detected and treated quickly [6]. The parasite species most frequently associated with transfusion malaria are *Plasmodium falciparum, P. malariae* and *P. vivax* [7, 8]. Blood donors with semi-immunity could harbor low levels of the *Plasmodium* parasites, which are mostly below the detection threshold of currently available assays. This will cause malaria parasites to persist for several years in infected blood recipients [7, 9]. During storage, malaria parasites have been found to survive in donated blood at temperatures between 2 °C and 6 °C for 3 weeks, with the estimated inoculum in transfusions from one to 10 parasites per donation [10, 11]. Transfusing one malaria parasite per microliter of infected blood, converts to about 500,000 infected red cells in one unit of blood [2]. Most reported cases of TTM were either through whole blood or red blood cell concentrates, with a few cases of TTM occurring after platelets and leukocytes transfusions [12].

Genetic diversity of *P. falciparum* has been attributed partly to its ability to evade host immune system [13] and adapt to anti-malarials [14]*.* Alleles of *P. falciparum* chloroquine resistance transporter gene (*Pfcrt*) have been associated with chloroquine resistance in *P. falciparum* [15]. *P. falciparum* multi-drug resistance (*Pfmdr*) gene is also associated with both chloroquine and amodiaquine resistance [16]. Mutations in *P. falciparum* dihydropteroate-synthetase (*Pfdhps*) gene confer resistance to sulfadoxine [17] while *P. falciparum* dihydrofolate-reductase (*Pfdhfr*) gene alleles are also associated with resistance to pyrimethamine [18]. High prevalence of mutations in *Pfdhfr* and *Pfdhps* will undermine the prophylactic effect of sulphadoxine–pyrimethamine in pregnant women and immigrants visiting malaria endemic regions from non-endemic countries [19]. Finally, non-synonymous single nucleotide polymorphisms in the *Kelch 13* propeller domain on chromosome 13 (*Kelch 13*) were found to be strongly associated with resistance to artemisinin derivatives [20].

Artemisinin-based combination therapies (ACTs) have contributed to the substantial decline in malaria burden. However, the efficacy of ACTs is threatened by the emergence of artemisinin resistance in *P. falciparum* that led to treatment failure of ACTs in South-East Asian countries namely, Thailand, Cambodia, Vietnam and Myanmar [21].

In previous studies in Ghana, high frequencies of mutation in *Pfcrt*, *Pfmdr1*, *Pfdhfr* and *Pfdhps* were identified in *P. falciparum* infected children aged 4–15 years [22, 23]. However, such study is yet to done in Ghanaian adults and for that matter, blood donors. Hence, this study was designed to determine the magnitude of the risk associated with the transmission of these putative anti-malaria drug resistant biomarkers through blood transfusion.

## Methods

### Study design

**Fig. 1:**
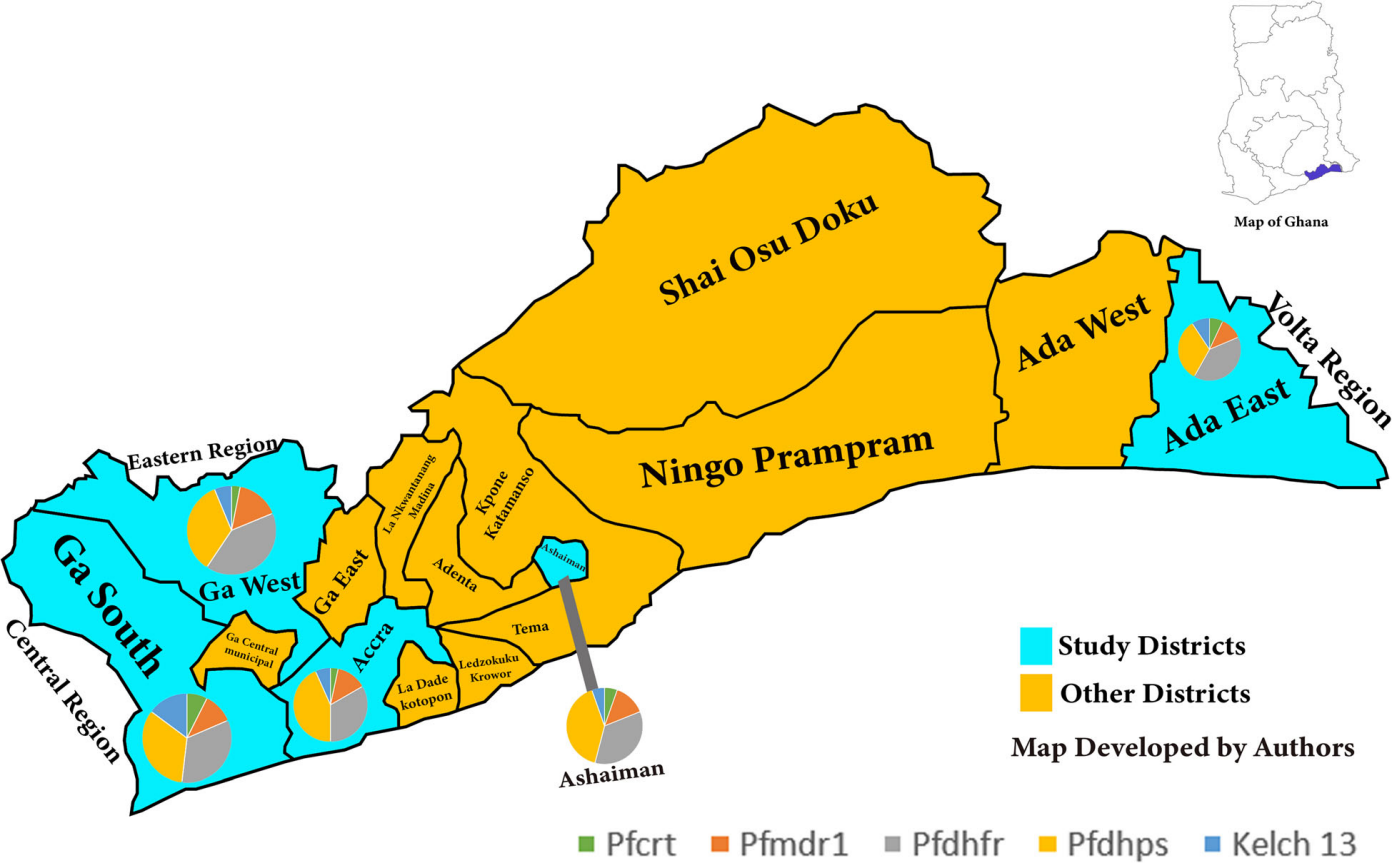
Map of Greater Accra region, Ghana showing study and other districts (map drawn by authors). The pie charts represent frequency of distribution of mutant alleles in each study site. The study districts were Accra Metropolis (5° 33′ 0″ N, 0° 12′ 0″ W), Ga West Municipal (5° 42′ 9″ N, 0° 18′ 0″ W), Ga South Municipal (5° 34′ 0″ N, 0° 20′ 0″ W), Ashaiman Municipal (5° 42′ 0″ N, 0° 2′ 0″ W) and Ada East District (5° 47′ 0″ N, 0° 38′ 0″ E)

This cross-sectional study involved consented blood donors that were randomly recruited from five districts in the Greater Accra region of Ghana. The districts were Ada East, Ashaiman, Accra Metropolis, Ga South and Ga West (Fig. 1). For each blood donor, 5 mL of CPDA-1 anticoagulated blood was collected, kept on ice packs (~ 6–10 °C) and sent to Ga West Municipal Hospital Blood Bank, Amasaman-Accra for analysis. Blood donors were recruited from November 2017 to August 2018. Each blood sample was screened for transfusion transmissible infections (TTIs) (HIV I&II, hepatitis B, hepatitis C and syphilis) using enzyme immunoassays as described previously [24]. All TTIs infected donor blood units were excluded from further analysis. The TTIs-negative donor blood units were then screened for malaria parasites. Subsequently, four dried blood spots were prepared on filter paper for all malaria infected donor blood units, and sent to Malaria Genome Laboratory, Wellcome Sanger Institute, Hinxton-United Kingdom for genotyping by sequencing for putative anti-malaria drug resistant biomarkers.

### Sample size determination

Minimum number of prospective blood donors recruited for this study was determined using the Cochrane’s formula: *n* = z^2^p(1-p)/d^2^, where *n* = sample size, p = proportion of blood donors infected with *P. falciparum*, 1-p = proportion of blood donors not infected with *P. falciparum, z* = confidence level at 95% (standard value of 1.96), d = margin of error at 5% (standard value of 0.05). Prevalence of *P. falciparum* in blood donors was determined in the Volta Region, the closest region to Greater Accra Region, be 10.0% [3]. Based on these values, the sample size was calculated to be 139 prospective blood donors.

### Donor selection

Blood donors were selected according to Medical History Guide for Donor Selection protocol [25]. Prospective donors included in the study were individuals aged between 18 and 59 years that had resided in their respective study districts for not less than 1 y. Consented blood donors were selected for this study by selecting any other donor that came to donate blood. This was done by sampling either all even or all odd numbered blood donors depending on the random choice for the donation day. Prospective blood donors were screened using National Blood Service, Ghana’s self-exclusive questionnaire. Additionally, donors’ malaria history was taken. In this study, donor selection and donor blood collection was done in collaboration with National Blood Service Ghana.

### Donor phlebotomy and study specimen collection

Donor phlebotomy was done according to Guidelines on drawing blood published by World Health Organization [26] with slight modification. Briefly, a large, firm vein, preferably in the antecubital fossa was selected. A tourniquet was applied to make the vein more prominent. Donor was made to squeeze the fist intermittently. Once the vein was selected, the skin site was disinfected using 70% isopropyl alcohol. The area was allowed to dry completely. Venipuncture was performed using a smooth, clean entry with 16-gauge needle which was attached to the blood collection bag. As soon as blood flow was established, the donor was asked to open and close the fist slowly every 10–12 s during collection whilst the tourniquet was removed. The donor was closely monitored during bleeding. About every 30 s during the donation, the collected blood was gently mixed with the anticoagulant using mechanical mixer. After adequate blood has been collected, the needle was removed, injection sites dressed, the needle was severed from the collected tube, blood labeled and kept on ice pack. Approximately 5 mL of anticoagulated blood sample was collected for laboratory testing.

### Study variables

The study variables were, completed age in years of blood donor, gender, number of successful previous blood donations, history of clinical malaria, present body temperature, malaria testing outcomes and frequencies of putative anti-malaria drug resistant alleles. Questionnaires were used to obtain the age, gender, number of successful previous donations and history of clinical malaria. However, body temperature was determined on the day of blood donation using digital infra-red non-contact thermometers (Kinlee, Guangdong). Malaria screening was performed with SD Bioline rapid diagnostic kit whilst putative anti-malaria drug resistant alleles were determined using DNA sequencing approach.

### Laboratory procedures

#### Screening for malaria parasites and parasitaemia determination

Donor blood malaria screening was done with the SD Bioline PfHRP-2/pLDH rapid diagnostic test kit (Gyeonggi-do, Republic of Korea). Malaria parasitaemia was determined in PfHRP2/pLDH positive donor units that satisfied the inclusion criteria. Malaria parasitaemia was quantified according to WHO protocol [27]. Quantification was done by dividing the number of asexual parasites per at least 200 leukocytes and multiplied by average WBC count of 8000 cells/μL of blood.

#### Preparation of dry blood spots (DBS)

Dried blood spots (DBS) were prepared using approx. 50 μL of anticoagulated whole blood. In all, four blood spots were prepared (two blood spots measuring approximately 15 mm in diameter on one filter paper). The blood spots were completely dried at room temperature.

#### *Plasmodium falciparum* genomic DNA extraction

*P. falciparum* DNA was extracted from dried blood spots (DBS) using QIAamp DNA Investigator Kit (Qiagen, California, United States) following kit manufacturer’s instructions. At least 5 ng of DNA was used as template for sequencing.

#### *Plasmodium falciparum* primers and probes used in this study

Primers used to identify and genotype *P. falciparum* genome in this study were developed by Oyola et al. [28] and previously used by Aninagyei et al. [24] to detect asymptomatic *P. falciparum* infections in blood donors. The primers were designed to bind to the *P. falciparum* genome reference genome, *P. falciparum* 3D7 genome.

#### ***P. falciparum*** genome sequencing and analysis

The detailed methodology for detecting and sequencing *P. falciparum* genome using selective whole genome amplification (sWGA) has been published by Aninagyei et al [24]. The sWGA reaction was performed using DNA template (≥ 5 ng). The reaction mix was made up of the following: template DNA, 1× bovine serum albumin, 1 mM dNTPs, 2.5 μM of each amplification primer, 1× Phi29 reaction buffer and 30 units of Phi29 polymerase enzyme (New England Biolabs). Isothermal amplification conditions were used (35 °C for 5 min, 34 °C for 10 min, 33 °C for 15 min, 32 °C for 20 min, 31 °C for 30 min, 30 °C for 16 h prior to denaturing Phi29 polymerase enzyme 65 °C. Ampure XP cleaned amplicons washed with 200 μL of 80% ethanol and eluted with elution buffer. Using the NEBNext® Ultra™ DNA library preparation kit (New England Biolabs), DNA libraries were prepared prior to sequencing on Illumina HiSeq 2500 DNA Sequencer. After sequencing, demultiplexing and fastq data files were generated automatically. Low quality reads were trimmed (Bioedit v7.2) and each dataset analysed independently by mapping sequence reads to the *P. falciparum* 3D7 reference genome using Burrows-Wheeler Aligner. Allelic analysis was done for *Pfcrt, Pfdhfr*, *Pfdhps*, *Pfmdr1* and *Kelch13* genes.

### Data processing and statistical analysis

All percentages were computed by simple proportions using Microsoft excel 2016. Association of malaria parasitaemia to total number of mutant alleles were done with Pearson correlation test while association between mutant alleles and demographic determinants were determined by Chi square test. All analyses were done with SPSS Version 24 (Chicago, IL, USA).

## Results

### Outcome of the donor recruitment and screening for asymptomatic *P. falciparum*

**Fig. 2:**
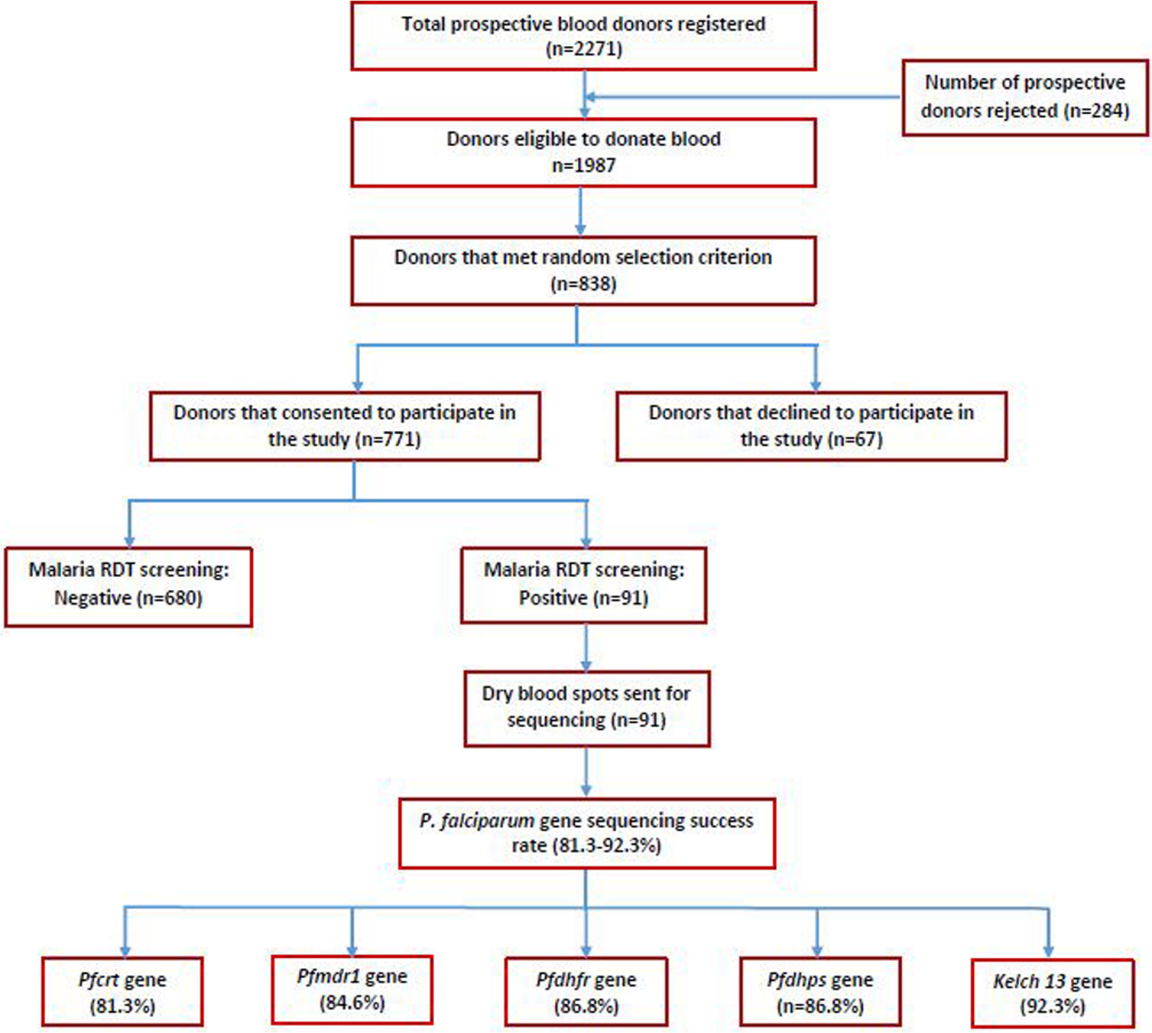
Flow chart for blood donor selection, *P. falciparum* screening and gene sequencing success rate. RDT-SD Bioline rapid diagnostic test kit, *Pfmdr1*- *P. falciparum* multidrug resistance, *Pfcrt*- *P. falciparum chloroquine resistance transporter*, *Pfdhfr*- *P. falciparum* dihydrofolate reductase, *Pfdhps*- *P. falciparum* dihydropteroate synthase, *Kelch 13*- *P. falciparum Kelch 13* propeller gene, n-number of donors or samples in each category

At the end of the donor recruitment period, the National Blood Service, Ghana registered 2271 prospective blood donors. Of this total, 1987 (87.5%) were eligible to donate blood based on the self-exclusion questions and haemoglobin concentrations levels. Random sampling method selected 838 prospective donors but 67 (8.0%) declined to participate in this study while consent was granted by 771 (92.0%) blood donors (Fig. 2). All blood donors were negative for transfusion-transmitted infections (HIV 1&2, syphilis, hepatitis B and C).

### Description and frequency of blood donations of the malaria infected blood donors

This study recruited 771 consented blood donors (aged 19–59 years). Of this number 91 blood donors representing 11.8% were found to be asymptomatically infected with *P. falciparum* parasite. The district with the most infected blood donors was Ada East district (18.1%) whilst Ga South municipal (9.2%) reported the least infected blood donors. Most of the infected blood donors were between 31 and 40 years and majority of them were males. More than half of the infected donors (64.8%) were donating for the first time whilst the rest had donated more than once. Eleven of the infected blood donors (12.1%) have had clinical malaria within the past year. Donor malaria parasitaemia did not associate past malaria episode within past year (*x*^*2*^ = 3.6, *p* = 0.059), number of blood donations (*x*^*2*^ = 8.2, *p* = 0.08) and gender (*x*^*2*^ = 2.3, *p* = 0.13). However, donor age associated with infection status (*x*^*2*^ = 18.8, *p* = 0.003) with higher infections observed in 31–40-year range. Meanwhile, the overall mean body temperature of the infected donors was 36.9 ± 0.56 °C (Table 1).

**Table 1:** District-wise demographic and clinical variables of the malaria infected blood donors

Demographic variables	Total *n* (%)	Ada East District (*n* = 149)	Ashaiman Municipal (*n* = 164)	Accra Metropolis (*n* = 155)	Ga South Municipal (*n* = 140)	Ga West Municipal (*n* = 163)
Malaria rapid test	91 (11.8)	27 (18.1%)	19 (11.6%)	15 (9.7%)	13 (9.2%)	17 (10.4%)
Age in complete years*						
18–30 (*n* = 171)	18 (19.7)	5 (18.5%)	3 (15.7%)	4 (26.7%)	2 (15.4%)	4 (23.5%)
31–40 (*n* = 268)	41 (45.0)	12 (44.4%)	9 (47.4%)	7 (46.7%)	5 (38.4%)	8 (47.0%)
41–50 (*n* = 238)	23 (25.3)	7 (25.9%)	5 (26.3%)	3 (20.0%)	5 (38.4%)	3 (17.6%)
51–59 (*n* = 94)	9 (9.9)	3 (11.1%)	2 (10.5%)	1 (6.7%)	1 (7.7%)	2 (11.7%)
Gender						
Males (*n* = 609)	78 (85.7)	22 (81.4%)	16 (84.2%)	13 (86.7%)	12 (92.3%)	15 (88.2%)
Females (*n* = 162)	13 (14.3)	5 (18.5%)	3 (15.8%)	2 (13.3%)	1 (7.7%)	2 (11.8%)
Number of blood donations						
1 (*n* = 395)	59 (64.8)	17 (62.9%)	13 (68.4%)	10 (66.7%)	8 (61.5%)	11 (64.7%)
2 (*n* = 124)	14 (15.4)	5 (18.5%)	2 (10.5%)	2 (13.3%)	1 (7.7%)	4 (23.5%)
3 (*n* = 111)	8 (8.8)	2 (7.4%)	2 (10.5%)	1 (6.7%)	2 (15.4%)	1 (5.9%)
4 (*n* = 89)	8 (8.8)	2 (7.4%)	1 (5.3%)	2 (13.3%)	2 (15.4%)	1 (5.9%)
> 5 (*n* = 54)	2 (2.2)	1 (3.7%)	1 (5.3%)	0 (0.0%)	0 (0.0%)	0 (0.0%)
At least one episode of malaria within the past 1 year						
Yes (*n* = 79)	11 (12.1)	5 (18.5%)	2 (10.5%)	0 (0.0%)	3 (23.1%)	1 (5.9%)
No (*n* = 692)	80 (87.9)	22 (81.5%)	17 (89.5%)	15 (100%)	10 (76.9%)	16 (94.1%)
Body temperature (°C)						
Mean ± standard deviation		36.7 ± 0.77	37.1 ± 0.12	36.88 ± 0.51	36.8 ± 0.68	37.0 ± 0.72
95% CI		36.1–37.40	36.8–37.4	36.4–37.3	36.1–37.6	36.3–37.6

* Significant association with donor parasitaemia

### *P. falciparum* genotype by sequencing success rate

The genotyping success rate for *P. falciparum chloroquine resistance transporter* (*Pfcrt*), *P. falciparum* multidrug resistance-1 (*Pfmdr1*), *P. falciparum* dihydrofolate reductase (*Pfdhfr*), *P. falciparum* dihydropteroate synthase (*Pfdhps*) and *P. falciparum Kelch 13* propeller gene (*Kelch 13*) genes are presented in Table 2. Of the 91 samples submitted for sequencing, the success rate ranged from 81.3% for *Pfmdr1* to 92.3% for *Kelch 13.* It was observed that sequencing was not successful for parasite count of less than 382 parasites/μL of blood (*n* = 7).

**Table 2:** *Pfcrt*, *Pfmdr1*, *Pfdhfr*, *Pfdhps* and *Kelch 13* genotyping success rate

Gene	Number of genes successfully genotyped	Percentage of genes successfully genotypes	Range of absolute parasite count (parasites/μL of blood)
*P. falciparum* multidrug resistance-1 (*Pfmdr1*)	74	81.3%	773–4487
*P. falciparum chloroquine resistance transporter* (*Pfcrt*)	77	84.6%	851–4487
*P. falciparum* dihydrofolate reductase (*Pfdhfr*)	79	86.8%	609–4487
*P. falciparum* dihydropteroate synthase (*Pfdhps*)	79	86.8%	609–4487
*P. falciparum Kelch 13* propeller gene (*Kelch 13*)	84	92.3%	382–4487

### Characterization of mutant haplotype identified in *Pfcrt*, *Pfmdr1*, *Pfdhfr*, *Pfdhps* and *Kelch 13* genes

In *Pfcrt* gene, mutations were only observed at amino acid positions 74, 75 and 76 but not at positions 72 and 73. At positions 74, 75 and 76, isoleucine, glutamic acid and threonine replaced methionine, asparagine and lysine respectively. The resultant haplotype was CVIET. These mutations occurred in only nine parasite isolates. In *Pfmdr1* gene, four different single nucleotide polymorphisms (SNPs) were identified. These SNPs resulted in different amino acids at positions 86 (tyrosine), 184 (phenylalanine) and 1246 (tyrosine) compared to the wild type. These nucleotide polymorphisms resulted in NFD, NYY, NFY and YFN haplotypes. The wild type haplotype of *Pfdhfr* gene is asparagine, cysteine, serine, isoleucine (NCSI) at 51, 59, 108 and 164 respective amino acid positions. Sequencing analysis indicated two different single mutations in the *Pfdhfr* gene. They were NRSI and NCNI where cysteine at position 59 mutated to arginine in the former and serine at position 108 changed to asparagine in the latter. Again, two double mutations resulted in NRNI at positions 59 and 108. Finally, three mutations in the *Pfdhfr* gene resulted in the haplotype IRNI where amino acid changes occurred in position 51, 59 and 108. Amino acid positions 436, 437, 540, 581 and 613 in the *Pfdhps* gene were found to be very polymorphic. The wild type haplotype for *Pfdhps* is SAKAA (S = serine, A = alanine and K = lysine). Single mutation at amino acid position 437 yielded one haplotype (SGKAA), double mutations at amino acid positions 436 and 437 yielded AGKAA haplotype. Three different triple mutation resulted in AGKAS (mutations at amino acid positions 436, 437 and 613), AGKSA (mutations at amino acid positions 436, 437 and 581) and FGKAS (mutations at amino acid positions 436, 437 and 613). One quadruple mutation resulted in AGKSS haplotype (mutations at amino acid positions 436, 437, 581 and 613) whilst one quintuple mutation resulted in AGESS haplotype (436, 437, 540, 581 and 613). Finally, five non-synonymous mutations were identified in the *Kelch 13* gene; I543V, A578S, P615L, C580Y and A676S. The frequencies of each mutant haplotype is presented in Table 3. The overall frequency of mutant haplotype in *Pfcrt*, *Pfmdr1*, *Pfdhfr*, *Pfdhps* and *Kelch 13* genes were 11.6, 29.7, 78.5, 87.3 and 16.6%. There was a moderate relationship between parasitaemia and frequency of mutant alleles (r = 0.42, *p* = 0.034).

**Table 3:** Frequencies of *P. falciparum* mutant gene haplotypes identified in blood donors

Gene	Total number of mutant haplotypes identified	Percentage mutant haplotypes identified
*Pfcrt* (*n* = 77)		
CVIET	9	11.6%
*Pfmdr1* (*n* = 74)		
NFD	4	5.4%
NYY	4	5.4%
YFN	6	8.1%
NFY	8	10.8%
*Pfdhfr* (*n* = 79)		
Single mutation		
NRSI	17	21.5%
NCNI	20	25.3%
Double mutation		
NRNI	6	7.6%
Triple mutation		
IRNI	19	24.0%
*Pfdhps* (*n* = 79)		
Single mutation haplotype		
SGKAA	13	16.4%
Double mutation haplotype		
AGKAA	12	15.2%
Triple mutation haplotype		
AGKAS	11	13.9%
AGKSA	9	11.4%
FGKAS	5	6.3%
Quadruple mutation haplotype		
AGKSS	5	6.3%
Quintuple mutation haplotype		
AGESS	14	17.7%
*Kelch 13* (*n* = 84)		
Non-synonymous mutations		
P615L	4	4.8%
A578S	4	4.8%
C580Y	3	3.6%
I543V	2	2.4%
A676S	1	1.2%

### Distribution of mutant alleles in the study sites

Almost equal number of *Pfcrt* CVIET haplotype was observed in Ada East, Ashaiman and Ga South while mutant *Pfmdr1* genes were almost equally distributed in the study areas. Also, majority of mutant *Pfdhfr* genes were seen in Ada East whereas, mutant *Pfdhps* genes were almost equally distributed. In *Kelch 13* genes, four non-synonymous SNPs were seen in Ada East and Ga South while two non-synonymous SNPs were seen in Ashaiman, Accra metropolis and Ga West (Fig. 1).

### Association of mutant haplotypes to demographic determinants

Chi square analysis indicated no association between mutant haplotypes of *Pfcrt*, *Pfmdr1*, *Pfdhfr*, *Pfdhps* and *Kelch 13* genes and demographic determinants. The distribution of mutant alleles was found to be comparable between all ages, gender and study sites.

## Discussion

In malaria-endemic countries, asymptomatic carriers of malaria parasites have been shown to exist and blood donors are likely to harbour the malaria parasites, though asymptomatically, which could later be transfused to patients who may require transfusion. Many of blood transfusion users are pregnant women, immunocompromised patients such as children under 5 years, HIV, TB, cancer and malnourished patients. These groups of people are particularly at higher risk of transfusion-transmissible malaria (TTM) which may be compounded by possible anti-malaria drug resistance strains.

This study therefore describes putative anti-malaria drug resistance biomarkers in blood donors that were recruited in the Greater Accra region of Ghana. The genes of interest were *Pfcrt*, *Pfmdr1, Pfdhps*, *Pfdhfr* and *Kelch 13* propeller genes. Allelic forms of these genes have been linked to anti-malaria drug resistance in several studies [15–18, 20].

In this study, the *Pfcrt* gene were found to contain three different amino acids at positions 74, 75 and 76 resulting in M74I, N75E and K76T. Importantly, K76T mutation has been implicated in chloroquine resistance [29, 30]. In Ghana, incidence of K76T has been reported in several studies [22, 31, 32], however, K76T mutation may not complicate TTM, since chloroquine is no longer the drug of choice for the treatment of malaria. In Ghana, the drug of choice for management of uncomplicated malaria is artemisinin-based combination therapy (ACT). Non-synonymous mutations in *Kelch 13* propeller gene is associated with artemisinin resistance especially in parasites causing infection in South-East Asia (SEA) [21].

In this study, five non-synonymous mutations P615L, A578S, I543V, A676S and C580Y were identified in the *Kelch 13* gene. The P615L mutation was detected in Pakistan [33]. Also, A676S has been identified in Africa and South-East Asia [34]. Again, S466N was identified in Peru [35] and subsequently in Colombia [36]. On the other hand, I543V allele has not been previously identified in any parasite strain. However, I543T, a very close allele to I543V, has been previously identified in SEA [34]. Outside SEA [34], C580Y has previously been identified in Africa specifically Cameroon [34] and Guyana in South America [37]. C580Y has been validated as artemisinin anti-malaria drug resistance allele [20, 34, 38–40], however, association of C580Y with anti-malaria drug resistance outside SEA has not been reported. *Kelch 13* A578S allele, as was observed in this study, has also been previously detected in Ghanaian parasite isolates. In Ghana, A578S has been previously identified in three regions, Eastern, Bono (formerly Brong Ahafo) and in the Upper West Regions [23]. The A578S allele is two codons away from C580Y allele. Malaria endemic regions that have previously identified A578S, must enhance surveillance for C580Y allele. Matrevi et al. [23] have also detected C580V (very close allele of C580Y) in Central region of Ghana. Furthermore, Feng et al. [41] in 2013 also detected three isolates of C580Y SNP in Chinese migrant workers from Ghana. Two years later (in 2015), Huang et al. [42] also detected some cases of C580Y from Ghanaian immigrants to China. These reports highlight the fact that C580Y allele is present in Africa and in Ghana in particular. It could, therefore, be concluded that C580Y allele could be an emerging *Kelch 13* SNP in Ghana. It is therefore suggested that enhanced surveillance for C580Y in Ghana be implemented to unearth the actual burden of the SNP.

Mutations in the *Pfmdr1* gene, have also been associated with chloroquine, amodiaquine, lumefantrine and mefloquine resistance [43, 44]. Amodiaquine, lumefantrine and mefloquine are artemisinin partner drugs. The most reported mutations in *Pfmdr1* gene globally is N86Y, Y184F and N1246Y [16, 40, 42, 43]. In Ghana, these alleles have also been identified to be associated with reduced sensitivity of ACTs and other anti-malaria mono-therapies [45]. This study identified the double mutant, Y184F/D1246Y represented as NFY haplotype in 10.8% of the isolates while the triple mutant, YFN (N86Y/Y184F/D1246N) was observed in 8.1% of the isolates. Two single mutants, NFD (Y184F) and NYY (D1246Y) were identified in 5.4% of the parasite isolates. Transmission of these haplotypes through blood transfusion, possibly will reduce the sensitivity of artemisinin partner drugs if infection is established. In Nigeria, N86Y and Y184F alleles have been associated with recurrent parasitaemia following Artemether-Lumefantrine therapy [46]. If even blood recipients are treated for malaria as suggested in other studies [47], they risk experiencing symptomatic infections due to the presence of N86Y and Y184F alleles.

In a study in the Volta region of Ghana, Kweku et al estimated malaria in pregnancy (MIP) to be 20.3% [48]. MIP is associated with severe maternal anaemia, low birth weight, increased perinatal mortality, pre-term birth, still birth and infant deaths [49, 50]. World Health Organization recommends intermittent preventive treatment of malaria in pregnancy (IPTp-MIP) with sulphadoxine-pyrimethamine (SP) to reduce the risk of poor MIP outcomes. With or without malaria parasitological testing, sulphadoxine-pyrimethamine for IPTp (IPTp-SP) is given to pregnant women. IPTp-SP is administered to either treat patient with parasites or provide a prophylactic effect to non-infected patients [51]. Mutations in two genes of *P. falciparum*, dihydropteroate synthase (*Pfdhps*) and dihydrofolate reductase (*Pfdhfr*) have been associated with resistance against sulfadoxine and pyrimethamine respectively. Accumulation of single point mutations in these genes have synergistic effect on reducing the efficacy of SP. The most important mutant biomarkers that increase the resistance of SP are combined triple *Pfdhfr* (N51I, C59R, S108N) and double *Pfdhps* (A437G, K540E) mutations resulting in quintuple mutation [52]. Analysis of genomic data obtained in this study indicated 24.0% triple mutations in the *Pfdhfr* gene (N51I, C59R, S108N resulting in IRNI haplotype) and 17.7% double mutations in *Pfdhps* gene (A437G, K540E resulting in AGESS haplotype). Further analysis revealed that 12 isolates representing 15.1% contained the quintuple mutations in the *Pfdhfr* and *Pfdhps* genes. This study reveal that quintuple mutations in the *Pfdhfr* and *Pfdhps* genes associated with infected donor blood could reduce the prophylactic efficacy of SP in pregnant women transfused with infected donor blood.

There is paucity of data with regards to previous findings on the prevalence and frequencies of anti-malaria drug resistant genes in blood donors in both endemic and non-endemic countries. However, several cases of anti-malaria drug resistant genes have been imported, by asymptomatic carriers, into non-endemic countries from endemic countries [41, 52–54]. Imported anti-drug resistant malaria is of public health concern since it has the potential to subvert global efforts to eradicate malaria. And also in regions with females *Anopheles* mosquitoes during transmission seasons, residents risk being transmitted with *P. falciparum* and more importantly, *P. falciparum* carrying drug resistant genes.

In a previous study [41], authors reported that imported *P. falciparum* malaria sharply increased in China, due to migrant workers who had returned from Ghana, a sub-Saharan malaria-endemic country. Of the 118 imported *P. falciparum* parasites that were evaluated, high prevalence of anti-malaria drug resistant gene polymorphisms, notably, N86Y, N86Y and D1246Y in *Pfmdr1* genes and K76T in *Pfcrt* were reported. Again, four non-synonymous mutations in *Kelch 13* gene were also identified (R539T, C580Y, C580F, D584V), one of which was identified in this study (C580Y). Findings from Feng et al. [41] and results obtained in this study confirm high prevalence of putative anti-malaria drug resistant biomarkers in asymptomatic infections in both Ghanaians and migrants from Ghana.

These findings raise concerns about the possible emergence of artemisinin resistance in Ghana. The impact of *Pfmdr1* and *Kelch 13* gene polymorphisms on Ghanaian *P. falciparum* parasite clearance times following ACT treatment need to be determined in further studies.

## Conclusion

This study underscores high prevalence of malaria in Ghana which translated into 11.8% of asymptomatic infections in blood donors. For the first time, this study reports the prevalence of putative anti-malaria drug resistant markers in blood donors. Detection of C580Y *Kelch 13* mutation, triple mutation haplotype, YFN, in *Pfmdr1* gene and quadruple mutations in the *Pfdhfr/Pfdhps* genes (*Pfdhfr*: N51I, C59R, S108N resulting in IRNI haplotype and *Pfdhps*: A437G, K540E resulting in AGESS haplotype) could contribute to ACT treatment failure and reduced efficacy of sulphadoxine-pyrimethamine (SP) in transfusion recipients of malaria infected blood. All blood donors living in malaria endemic regions should be screened for malaria parasites to avert possible transmission of anti-malaria drug resistant genes to blood recipients.

### Limitations of this study

Inability to obtain 100% gene sequencing success rate could affect the relative frequencies and the distributions of the *P. falciparum* anti-malaria drug resistant gene alleles. However, results obtained in this study underscore the fact that anti-malaria drug resistant genes exist in Ghana.

## Data Availability

Datasets generated and analysed in this study are available in the Harvard data verse repository (https://dataverse.harvard.edu/dataset.xhtml?persistentId=doi:10.7910/DVN/4ZZ0EY). However, a request for the data can be obtained from the corresponding author on reasonable request.

## Abbreviations

ACT: Artemisinin-based combination therapy
DBS: Dried blood spot
IPTp-MIP: Intermittent preventive treatment of malaria in pregnancy
*Kelch 13*: Kelch 13 propeller domain on chromosome 13
MIP: Malaria in pregnancy
*Pfcrt*: *P. falciparum* chloroquine resistance transporter
*Pfdhfr*: *P. falciparum* dihydrofolate-reductase
*Pfdhps*: *P. falciparum* dihydropteroate-synthetase
PfHRP2: *P. falciparum* histidine rich protein 2
*Pfmdr*: *P. falciparum* multi-drug resistance
*pLDH*: *Plasmodium* lactate dehydrogenase
RDT: Rapid diagnostic test
SNPs: Single nucleotide polymorphisms
sWGA: Selective whole genome amplification
TTI: Transfusion transmissible infection
TTM: Transfusion transmissible malaria
AGKAA: Alanine-glycine-lysine-alanine-alanine
AGKAS: Alanine-glycine-lysine-alanine-serine
AGKSA: Alanine-glycine-lysine-serine-alanine
AGKSS: Alanine-glycine-lysine-serine-serine
CVIET: Cysteine-valine-isoleucine-glutamic acid-tyrosine
FGKAS: Phenylalanine-glycine-lysine-alanine-serine
NCNI: Asparagine-cysteine-asparagine-isoleucine
NCSI: Asparagine-cysteine-serine-isoleucine
NFD: Asparagine-phenylalanine-aspartic acid
NFY: Asparagine-phenylalanine-tyrosine
NRSI: Asparagine-arginine-serine-isoleucine
NYY: Asparagine-tyrosine-tyrosine
SAKAA: Serine-alanine-lysine-alanine-alanine
SGKAA: Serine-glycine-lysine-alanine-alanine
YFN: Tyrosine-phenylalanine-asparagine
